# Health-related quality of life of patients with zygomatic fracture

**DOI:** 10.4317/medoral.21914

**Published:** 2017-08-16

**Authors:** Leena Kaukola, Johanna Snäll, Risto Roine, Harri Sintonen, Hanna Thorén

**Affiliations:** 1 Department of Oral and Maxillofacial Diseases, University of Helsinki and Helsinki University Hospital, Helsinki, Finland; 2 University of Eastern Finland, Research Centre for Comparative Effectiveness and Patient Safety, Department of Health and Social Management, Kuopio, Finland; and Helsinki and Uusimaa Hospital District, Administration, Helsinki, Finland; 3 Department of Public Health, University of Helsinki, Helsinki, Finland

## Abstract

**Background:**

The objective was to evaluate health-related quality of life (HRQoL) before and after surgical treatment of zygomatic complex fracture and assess patients’ perceptions of the aesthetic and functional outcomes of surgery.

**Material and Methods:**

A prospective study of 79 adult patients before and after surgery for zygomatic complex fracture was conducted. HRQoL was measured using the generic 15-dimensional (15D) instrument, and patient satisfaction was assessed by an additional questionnaire.

**Results:**

The mean preoperative 15D score for patients was lower than for general population that was matched for age and gender (*p*=0.011). The mean 15D score was lowest on the first postoperative day (*p*<0.001) when patients were worse off for 6 of the 15 dimensions of the HRQoL instrument and better off for three dimensions. However, patients achieved, and even exceeded, the mean 15D score of the general population during the first month following surgery. Infraorbital sensory loss at the end of the six-month follow-up appeared to be the single most important factor that plagued the patients.

**Conclusions:**

HRQoL is significantly reduced after trauma but improves a few weeks after surgery. Infraorbital nerve sensory loss is a notable long-term factor that affects patients after zygomatic complex fracture.

** Key words:**Zygomatic fracture, maxillofacial trauma, health-related quality of life, disturbance of infraorbital nerve, facial sensation.

## Introduction

Of all the facial bones, the zygoma is particularly susceptible to trauma because of its prominent position in the upper lateral midface. The zygomatic buttress as such is a strong, thick bone but the multiple sites of articulation with adjacent facial bones are vulnerable to external forces. Zygomatico-orbital fractures are often observed in patients with facial injuries, and are the most common fracture type after mandibular fractures (1,2).

The zygoma constitutes part of the orbital floor and the lateral wall of the orbit and is in close proximity to the infraorbital nerve that supplies sensory innervation to the face and parts of the oral cavity. The zygoma also serves as a site of attachment for facial mimetic muscles and masticatory muscles. Because of the central location of the zygoma near the crucial structures of face, a variety of problems may arise after zygomatic fracture in addition to oedema and pain after trauma and surgery. Previous studies have reported consequences such as changes in masticatory function and limited mouth opening (3), and infraorbital nerve dysfunction (3-6). Aesthetic impairments, e.g. facial scarring and asymmetry of the face, should also be considered as factors that affects patient health and wellbeing.

Apart from minor zygomatic bone fractures that heal without intervention, trauma surgery is often needed. Surgical fixation of the zygomatic complex fracture is often necessary for rehabilitating facial contour and achieving a good aesthetic outcome. Effective recovery of the impaired functions and sensation of the face often require surgery as well. In addition studies of facial trauma have reported that patients with sustained facial fractures have a higher incidence of social, psychological and behavioural problems (7-10) and are at risk of diminished health-related quality of life (HRQoL) (11,12). Yet, there are no previously published studies on the topic of HRQoL related to zygomatic trauma. We, therefore, decided to perform a six-month prospective follow-up study to evaluate patients HRQoL before and after surgical treatment of a zygomatic complex facture. HRQoL was studied by means of the 15D, which is a multidimensional generic HRQoL instrument. An additional structured self-report questionnaire was also used to find out patients’ perceptions about the recovery.

## Material and Methods

- Patient selection

Patients who were at least 18 years of age and needed surgical treatment of a zygomatic complex fracture (i.e. tripod fracture) were included in the study. Patients who underwent reconstruction of the orbital wall were excluded. The recruitment of the subjects was carried out during a four-year time period and patients were followed up for 6 months postoperatively.

- The 15D instrument

The HRQoL of the patients was assessed by the 15D instrument that is a standardized, comprehensive measure of HRQoL. The 15D questionnaire consists of 15 dimensions: mobility, vision, hearing, breathing, sleeping, eating, speech, excretion, usual activities, mental function, discomfort and symptoms, depression, distress, vitality and sexual activity. Each dimension was categorized into five non-overlapping levels that ranged from no problems to severe problems. The respondents were allowed to choose the level that best describes their current health status by ticking the appropriate box. These individual values were then converted into dimension level values and single index scores (15D scores) using a set of population-based preference or utility weights (13). The 15D score represents the overall HRQoL of the patients and ranges from the highest score of 1 (representing full health) to the minimum score of 0 (equivalent to being dead). The dimension level values reflect the patient’s perception for how he/she perceived the dimension expressed as a numerical score over the entire scale. The dimension level values and 15D scores of the patients were compared with those of an age- and gender-standardized sample of the general Finnish population. A change of approximately ±0.015 in the 15D score is considered clinically important because people can usually sense such a magnitude of change (14).

The 15D questionnaire was given to the patients for self-administration at the hospital before surgery and postoperatively during each of the clinical follow-up appointments on 1 day, 2 days, 1 week, 1 month, 3 months, and 6 months after surgery. When the patient did not attend the follow-up visit, the questionnaire was left uncompleted.

- Additional questionnaire

We designed a structured self-completion questionnaire in order to gain more detailed information about patients’ perceptions concerning their recovery and to find out how satisfied the subjects were with the aesthetic and the functional outcomes of the surgery. Patients completed the questionnaire during the follow-up visits that took place every 1, 2 and 3 months after surgery. As with the 15D, the questionnaire remained uncompleted, when the respondent did not attend the follow-up appointment. The questionnaire consisted of 7 multiple-choice questions except for the 6 month assessment which included two additional questions. The questionnaire explored patients´ perceptions regarding facial appearance, sensation, occlusion, chewing, and overall recovery. For each of these parameters, the respondents chose from the following levels the one that best described their current health status: 1) bad outcome, 2) moderate outcome, and 3) good outcome.

- Statistical analyses

Data were analyzed using the SPSS for Windows statistical software version 19.0 (SPSS, Inc., Chicago, IL, USA). The results are given as means with standard deviations (SD). The statistical significance of the differences between baseline and follow-up HRQoL scores was analyzed using paired samples t-test and the differences between the patients and the general population with the independent samples t-test. P-values less than 0.05 were considered statistically significant. The data for the general population were obtained from the National Health 2000 Health Examination Survey, which covers a sample of the Finnish population aged 30 years and older 15 and was matched for age and gender of the patients.

- Ethics committee approval

The Ethics Committee of the Department of Surgery and the Internal Review Board of the Division of Musculoskeletal Surgery, Helsinki University Central Hospital, Finland, approved the study (Dno 33/E6/06). Informed written consent was obtained from all participating patients.

## Results

The original study population comprised 81 patients of which 79 agreed to participate. The sex distribution within the group was 77% (61) male and 23% (18) female. The response rates at the various follow-up points varied for the 15D between 63% (50 patients) and 97% (77 patients) and for the additional questionnaire between 63% (50 patients) and 82% (65 patients) over the six-month follow-up period.

 Table 1 summarizes how the injury occurred. The most common cause of zygomatic fracture among women was falling, which accounted for half of the cases. Men were most likely to have zygomatic fracture as a consequence of an assault. The left side of the face was affected more often (61% of the cases) than the right side. Alcohol was involved in 42% of the incidents.

**Table 1:**
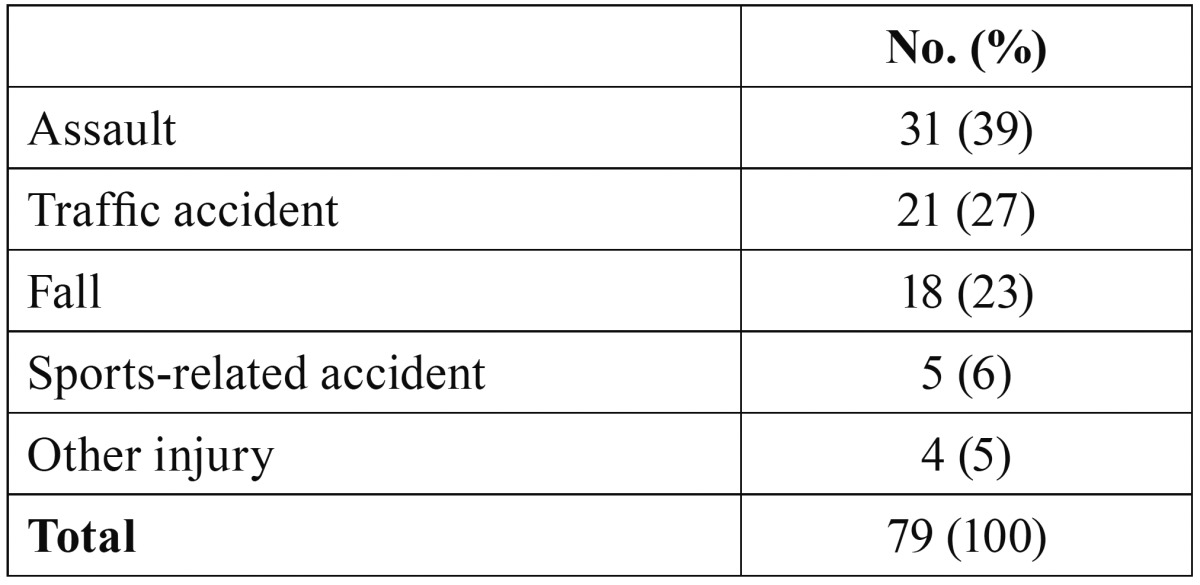
Mechanisms of injury.

Fracture reduction was performed with the aid of the Gillies approach, with the aid of a hook that was inserted under the zygomatic arch through a small buccal skin incision, or with the aid of an intraoral approach. In order to achieve optimal stability of the displaced fractures, 1 to 3 titanium miniplates were used for the fixation in the majority of the patients. The surgical approaches to the fracture lines varied between the following: upper eyelid blepharoplasty, lower eyelid blepharoplasty, transconjunctival approach, frontozygomatic suture approach or intraoral approach.

 Table 2 summarizes the surgical approaches and fracture reduction techniques that were used. The mean treatment delay, measured from the moment of the trauma to the surgical procedure, was 5.9 days. The mean preoperative 15D score of the patients (0.911) was significantly worse than that of age- and gender-standardized general population (0.932) (*p*=0.011).

**Table 2:**
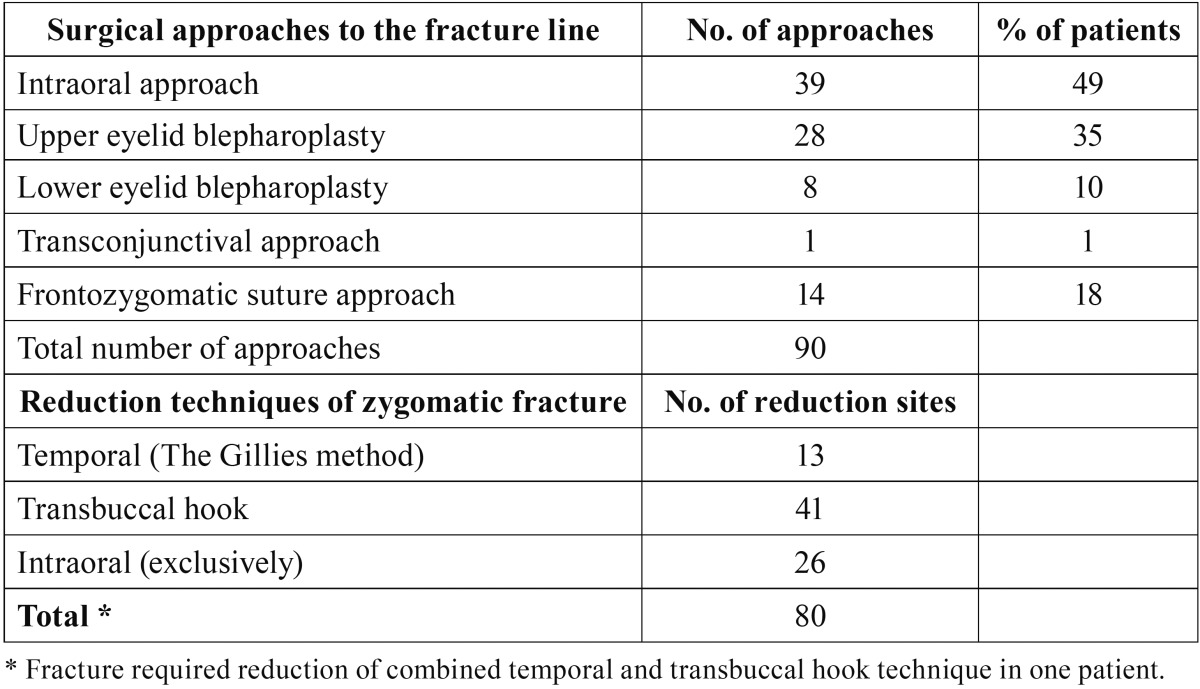
Surgical approaches to the fracture line and repositioning techniques of zygomatic complex fractures in 79 patients.

**Figure 1:**
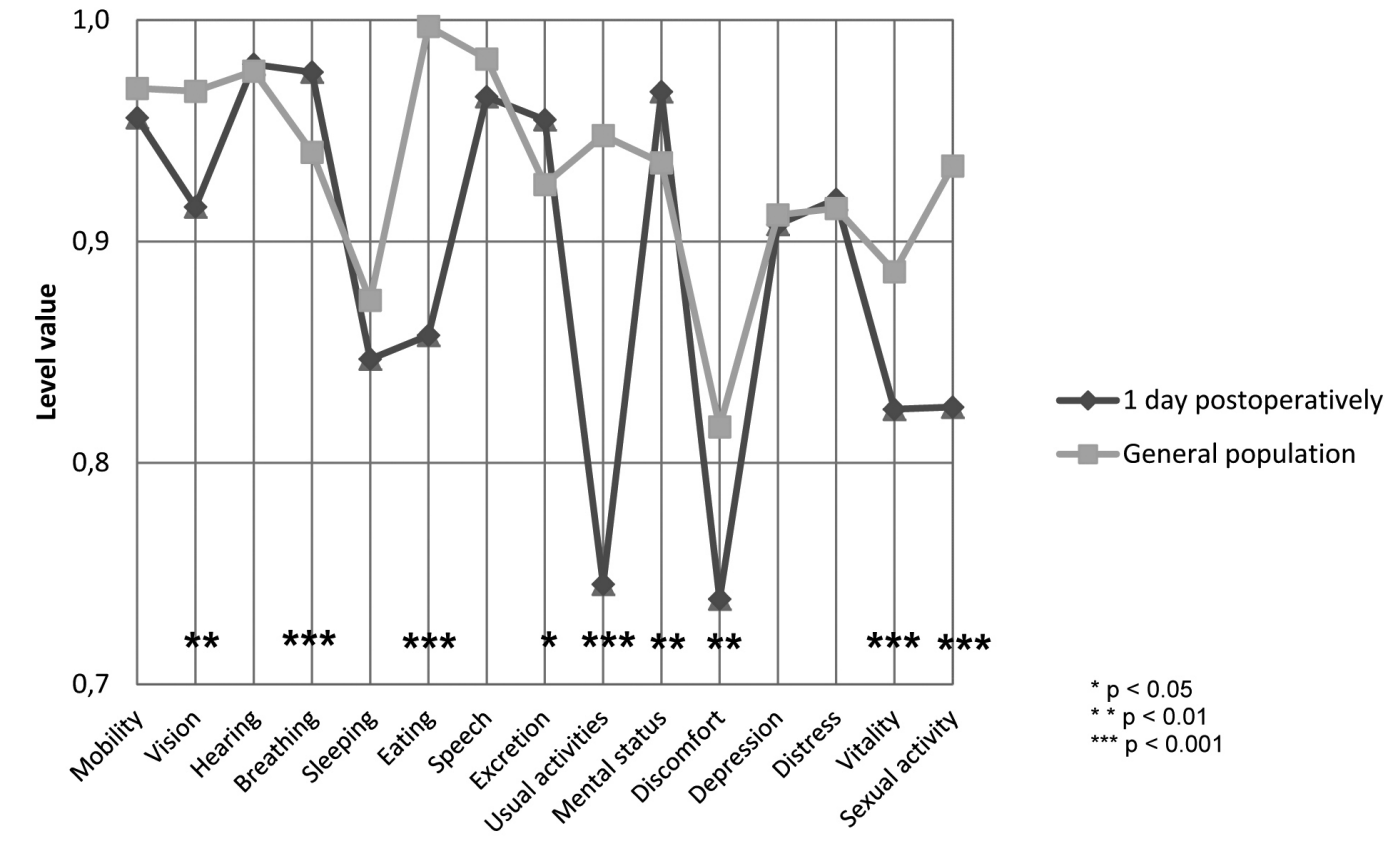
A comparison of the mean 15D profile of the patients on first postoperative day with those of the age- and gender-standardized general population.

**Figure 2:**
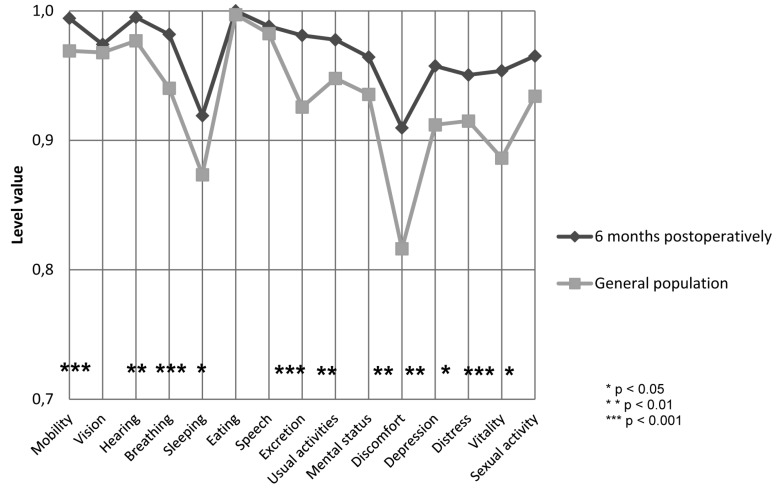
A comparison of the mean 15D profile of the patients at 6 months postoperatively with those of the age- and gender-standardized general population.

**Figure 3:**
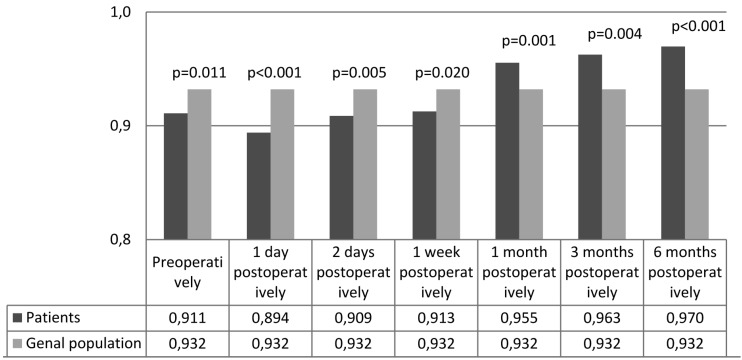
A comparison of the mean 15D scores at the follow-up appointments with those of the age- and gender-standardi-zed general population.

Figure 1 shows the mean 15D profile at the first postoperative day relative to that of the general population. Patients were significantly worse off for the six dimensions of vision (*p*=0.001), eating (*p*<0.001), usual activities (*p*<0.001), discomfort and symptoms (*p*=0.002), vitality (*p*=0.001) and sexual activity (*p*<0.001), and better off for breathing (*p*<0.001), excretion (*p*=0.025) and mental function (*p*=0.009). Figure 2 demonstrates the mean 15D profile of the patients 6 months postoperatively relative to the general population. Figure 3 shows the mean 15D scores of the patients at each follow-up appointment relative to the general population. After the surgery, the mean 15D score of the patients decreased to the lowest level on the first postoperative day. Up to one week postoperatively the mean 15D score of the patients (0.913) remained significantly lower than that of general population (*p*=0.020), but subsequently rapidly improved. At one month postoperatively the mean 15D score of the patients (0.955) had even exceeded that of age- and gender-standardized general population, the difference was statistically significant (*p*=0.001) and clinically important. Thereafter, the mean 15D score of the patients remained higher than that of the general population until the end of the 6-month follow-up period.

**Figure 4:**
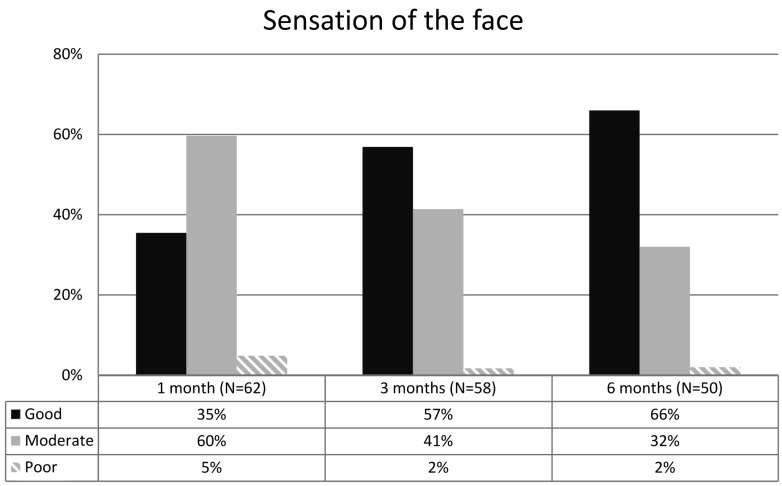
Patients (N=50-62) subjective opinnions about facial sensation at 1, 3 and 6 months after surgical fixation of zygomatic fracture.

A disturbance of the facial sensation was the most common complication resulting from zygomatic fracture although improvement was seen during the follow-up period. Immediately after the trauma 70 patients (89%) were diagnosed with impaired facial sensation in the region of the infraorbital nerve. Three months postoperatively 46 patients were suffering from sensory loss and at the end of the six-month follow-up period 27 patients were still left with sensory impairment of the face. Figure 4 illustrates the improvement of facial sensation after surgical treatment of a zygomatic fracture as perceived by the patients. Only 66% of the patients considered their facial sensation as good.

Patients´ perceptions of facial appearance, occlusion, chewing, diplopia and overall recovery improved over the six months of follow-up. Problems regarding aesthetics were of little significance in our study. Zygomatic trauma and the surgical treatment of the fracture caused only minor cosmetic defects since 98% of the patients considered their facial appearance to be good or moderate 6 months postoperatively. None of the patients considered having bad scars due to surgery. Twenty-eight patients (43%) reported eating problems and seven patients (11%) suffered from malocclusion one month after the surgery. Six months postoperatively only two patients (4%) reported minor eating problems and 1 patient (2%) had problems with occlusion. Diplopia was reported by five patients (7%) after the surgery, four of whom recovered within 3 months. None of the patients ended up with permanent diplopia. Overall recovery, which took into account all the aspects of the healing process, was considered good by 94% of the patients.

## Discussion

The main purpose of this study was to measure HRQoL before and after surgical fixation of a zygomatic fracture. The 15D instrument was used for the measurement of HRQoL. We also evaluated patients’ perceptions of the respective recovery by requiring them to complete an additional self-completed questionnaire.

The results of the study confirmed that patients suffered from significantly lower HRQoL after zygomatic fracture compared to that of the general population. Patients were significantly worse off for six of the 15 dimensions and better off for two. Until the first postoperative week the mean 15D score of the patients remained significantly lower than that of the general population. However, between one week and one month the scores began to improve and one month postoperatively patients scored even higher than the general population. This difference was both clinically important and statistically significant. The patients’ subjective opinions concerning most aspects of the recovery were positive over time, however, facial sensation was considered good by only 66% of the patients at the end of the six-month follow-up period.

Previous studies have reported that patients with zygomatic fracture suffer from problems such as infraorbital nerve paresthesia (3,4,16), visual problems (16), limited mouth opening and malocclusion (16). Pain, bruising, scarring, oedema and facial deformity were also found to be common after trauma. Results of our study support all of those findings. We also found that patients with a zygomatic fracture exhibited not only physical characteristics but also symptoms and signs of psychological impairment in some dimensions. Vitality, which is one of the psychological dimensions of the 15D was considerably affected among our patient population. Our finding is in line with those of previous studies, which reported that patients with facial trauma manifest post-traumatic psychiatric symptoms and social problems that significantly reduce their overall HRQoL. (11,12). It is worth noting that assaults caused a major part (39%) of the fractures in this study and it is plausible therefore that the victim in addition to the aggressor were already manifesting psychological behaviours that were exacerbated by alcohol at the time of the assault. However, unlike the findings reported by Ukpong *et al.* (11), we did not find any long term changes in the domains of psychological health of HRQoL. All the physical and mental dimensions of 15D improved quickly after the trauma and even exceeded that of age- and gender-standardized general population. Thus, according to the findings of our study it seems that the negative impact of a zygomatic trauma on patients’ HRQoL was only temporary.

The finding that patients with zygomatic fracture reported better HRQoL than healthy controls could be explained by the fact that people usually tend to compare their level of satisfaction with life in relation to earlier events and come to appreciate health more after a traumatic experience. Educational factors could also have affected the results. Patients in this study were recruited from Helsinki University Hospital (HUH) area, where the overall level of education is higher compared to the rest of Finland. Higher level of education has been observed to be associated with a better HRQoL.

Infraorbital nerve injury is a commonly reported complication after zygomatic trauma. Many previous studies have reported infraorbital nerve defects after complex zygomatic fractures (4-6). The sensory disturbance found in our study was the most common and disturbing impairment after zygomatic fracture. We studied 79 patients with zygomatic complex fractures of whom 89% were diagnosed with sensory loss. Dislocated zygomatic bones commonly compress the infraorbital nerve causing a nerve injury. Depending on the severity of the injury to the nerve, symptoms usually range from transient paresthesia to prolonged or even permanent numbness in the distribution area of the infraorbital nerve. The infraorbital nerve innervates the side of the nose, lower eyelid, cheek, upper lip, parts of the gingiva and some teeth, thus these areas are affected when the nerve is damaged. The findings of our study concerning infraorbital nerve recovery after zygomatic trauma were similar to those of other related studies that have reported neural improvement to last from weeks to several months depending on the severity of the nerve damage (5,6). However, we did not study neural recove-ry after 6 months even if it is likely that infraorbital nerve recovery still occurs after that time point.

In summary, zygomatic fracture patients’ quality of life is momentarily impaired after trauma and surgery, but improves rapidly during the first month to normal levels. Assessment of patients’ mental status and well-being is important during the first few weeks post-injury. Sensory loss in the area of the infraorbital nerve remains in most patients as a long-term disadvantage after the fracture. However, this does not appear to affect the overall quality of life.
